# “We are running on the fumes of goodwill” Professionals’ experiences of delivering 24/7 end-of-life care to children and their families: a qualitative study

**DOI:** 10.1186/s12904-025-01958-1

**Published:** 2025-12-03

**Authors:** Laura Barrett, Lorna Fraser, Lucy Ziegler, Stuart Jarvis, Susan Picton, Julia Hackett

**Affiliations:** 1 Department of Health Sciences, Faculty of Science, University of York, Seebohm Rowntree Building Heslington, York, YO10 5DD UK; 2 Cicely Saunders Institute, King’s College London, London, UK; 3 Academic Unit of Palliative Care, University of Leeds, Clarendon Way, Leeds, UK; 4 Leeds Teaching Hospitals NHS Trust, Beckett Street, Leeds, UK

**Keywords:** Paediatric end of life care, Paediatric care services, Palliative care, Qualitative research, Guidelines, Care pathways

## Abstract

**Background:**

Despite recent improvements, there is still stark inequity in the funding and provision of 24/7 end-of-life care for children, resulting in many families not receiving the support they need. To inform, plan and implement service changes it is important to take account of what works in current contexts and existing models of care, and to learn how professionals ‘on the ground’ are currently experiencing 24/7 care delivery.

**Methods:**

The study aimed to explore professionals’ perspectives of delivering 24/7 paediatric palliative care and their expectations and needs of a new service. This qualitative study used focus groups, and a thematic framework approach to analyse the data. Participants were healthcare professionals (HCPs) involved in the delivery of care to children (0–18 years) with palliative care needs and their families.

**Results:**

Fifty-three healthcare professionals, (25 doctors, 19 nurses, 6 managerial/administration and 3 allied professionals), took part in 11 focus groups. Three themes with sub-themes were developed: (1) Working within a fragmented landscape (Responding to the need for 24/7 end-of-life care, Coordination across teams without infrastructure, Building 24/7 continuity through integration); (2) Constraints on choice: default not preferred choice? (Limits to family choice, Critical yet inconsistent provision of community nursing, Inequality of access to specialist support); and (3) The personal cost of making it work (Gaps in confidence and experience, The price of goodwill). Professionals navigated a disjointed system to deliver 24/7 care to families. They strived to offer care in families’ preferred place; however, choice was constrained by the availability of local services. Professionals stretched themselves to provide around the clock care, often sacrificing their personal wellbeing and in doing so, inadvertently sustaining a broken system.

**Conclusion:**

Stepping up to support families with 24/7 end-of-life care for their child, has resulted in an unsustainable physical and emotional toll on professionals. The impact of delivering care in an inequitable system is causing significant moral distress, and there is a growing realisation that their goodwill is masking current systemic shortcomings. Integrated Care Boards must work jointly to find economies of scale to establish equitable and sustainable models of delivery that meet national standards and to ensure all children have access to high quality 24/7 end-of-life care.

**Supplementary Information:**

The online version contains supplementary material available at 10.1186/s12904-025-01958-1.

## Key statements

**What is already known about this topic?:**

• Providing 24/7 access to professionals with palliative care knowledge is key to supporting children and their families receiving end-of-life care in their preferred place, however there is stark inequity in funding and provision, resulting in many children and their families not receiving the care they need.

**What this study adds:**

• Regional service variations meant professionals were reactively finding ad hoc solutions for families; without supporting infrastructure in place, it takes time and resources to coordinate 24/7 end-of-life care across multiple teams.

• Professionals experienced moral distress when inequality in service provision constrained their ability to offer some families choice of place of end-of-life care.

• The study showed that sustaining 24/7 end-of-life care often relied on the goodwill of overstretched professionals leading to emotional and physical strain and concerns their efforts were masking systemic shortcomings.

**How this study might affect research, practice or policy:**

• ICBs need to work jointly to develop equitable and sustainable models of 24/7 end-of-life care to meet statutory duties and to ensure all children have access to high-quality care at the end of their lives.

**Supplementary Information:**

The online version contains supplementary material available at 10.1186/s12904-025-01958-1.

## Background

Families with a child nearing the end of life should have access to individualised palliative care including choice of preferred place of care [1–3]. This is clearly recommended in national and international quality standards and guidelines [4–8] as well as academic research [9]. In England, over 3,500 children die every year [10], and evidence has shown the importance to many parents of their child’s end-of-life care being delivered at home for as long as is possible [11, 12]. Changing symptoms, pain or parents’ distress cannot wait for ‘opening hours’ [13], so providing 24/7 access to professionals with palliative care knowledge is key to supporting this choice [3, 9, 12, 14]. 

In the UK, there has been little central planning or coordination of paediatric palliative care, and services have developed locally with different care models, a range of providers and variable integration with the hospice sector [15, 16]. Most hospices are primarily charity-funded and independently run, receiving around a third of their income from the NHS and central government [17]. The Health and Care Act 2022 places a legal obligation on the 42 Integrated Care Boards (ICBs) across England to commission palliative and end-of-life care for all age groups in their local areas, yet only about half of all ICBs have developed a service specification for children’s palliative care [8]. Despite recent improvements it is widely acknowledged there is still stark inequity in the funding and provision of 24/7 end-of-life care for children [18], resulting in many children and their families not receiving the care they need [8]. 

There is limited published evidence on best practice models to provide out-of-hours end-of life care for children and their families [9]. Calls for increased resources and improved leadership should, in time, lead to more consistent and equitable services [8], but to inform, plan and implement service changes it is important to understand what works in different local contexts and existing models of care, and to learn how professionals ‘on the ground’ currently experience the delivery of 24/7 end-of-life care.

This workstream is part of a wider study aimed at supporting the planning and provision of 24/7 paediatric palliative care in one of the seven NHS English regions, comprising four ICBs. The workstream’s aim was to explore professionals’ perspectives of delivering 24/7 paediatric palliative care and their expectations of a new service. Results from other workstreams of the study, including parents’ experiences are reported elsewhere [19].

## Methods

This was a qualitative study using focus groups and framework analysis. An interpretivist approach was taken to understand the subjective meaning of a range of healthcare professionals’ perspectives of delivering 24/7 care, whilst recognising the importance of researcher influence [20, 21]. The study is reported in accordance with Consolidated Criteria for Reporting Qualitative Research (COREQ) guidelines [22].

### Patient and Public Involvement

A parent advisory panel of five parents with diverse experiences, advised throughout the study, from application development to dissemination. Input included advice on recruitment strategy, development of topic guides, analysis and interpretation.

### Ethics

Ethical approvals were obtained from the Health Research Authority and Health and Care Research Wales (21/10/2022, IRAS 317352).

###  Population

The study was conducted across four ICBs in one region of England. Participants were healthcare professionals (HCPs) involved in the delivery of care and support to children (0–18 years) with palliative care needs, and their families. These included professionals with specialist palliative care experience: NHS specialist paediatric palliative care teams; paediatric oncology outreach teams and hospice teams. Also eligible were children’s community nurses; community-based paediatricians; general practitioners; hospital-based paediatric doctors and nurses; and allied health professionals.

### Sampling and recruitment

Professionals were identified and approached by palliative care teams via four NHS and six hospice sites. Participants were purposively sampled according to their team, role and geographic location and provided with an electronic brief information sheet. Interested participants contacted the research team directly by email and were sent a full information sheet.

### Data collection

Focus groups were conducted, with participants organised by professional role, to enable comparisons of 24/7 care provision across the region. Separate groups were run for NHS specialist palliative care teams, hospice-based teams, community professionals, paediatric professionals working in tertiary sites and those based in local district general hospitals.

All focus groups were conducted online via video call by two authors LB and JH (both females, applied health researchers, and previously unknown to participants). Participants completed an electronic consent form. The groups were asked about their roles in delivering end-of-life care for children, their experiences of 24/7 care delivery, what worked well and less well, and suggested improvements for families (Supplementary file 1). Focus groups were recorded and transcribed. Transcriptions were not returned to participant for checking.

### Data analysis

Analysis followed Gale et al.’s seven-step framework method: transcription, familiarisation, coding, developing the framework/codebook, applying the framework, charting data into the framework and interpretation [23]. LB read and reread the focus group transcripts for data familiarity and notes were taken on key concepts. Initial coding by LB, in Excel, used a deductive framework based on the discussion guide but also allowing for new inductive codes to capture unexpected or novel perspectives. Analysis was conducted alongside data collection, and the framework then developed iteratively through team discussion by combining the inductive and deductive codes into categories. LB applied this framework to all focus group transcripts, and once coded, the data were charted. This involved summarising the data in a spreadsheet and including key or illustrative quotes. Concepts and patterns in the data were then identified and discussed with the wider team to form themes.

### Reflexivity statement

Throughout the research process, the existing knowledge and experience of the research team was acknowledged. The team included applied health researchers with deep expertise in conducting sensitive qualitative research and clinicians with experience of paediatric palliative care. Input from the wider team and external stakeholders, including the parent advisory panel, offered diverse perspectives and helped establish a more gender-balanced team. Ongoing critical reflection took place throughout the study, particularly in relation to the chosen methods, development of the topic guide, and the analysis and interpretation of the findings.

## Results

Fifty-three healthcare professionals (HCPs) from 21 services in the region participated in 11 focus groups. See Table 1 for details of participant characteristics.

**Table 1:** Participant characteristics

Participant characteristics (*N* = 53)	
Participants per setting type	
Tertiary centre	20
District General Hospital	9
Community setting	11
Hospice	13
Participants by service type	
NHS Specialist Paediatric Palliative Care	12
Hospice	13
Paediatric Oncology Care Team	6
Children’s Community Nursing and GPs	9
General and Other Specialist Paediatricians	13
Participants by role	
Doctor	25
Nurse	19
Managerial/Administration	6
Family Support and Allied Professionals	3

### Themes

**Fig. 1:**
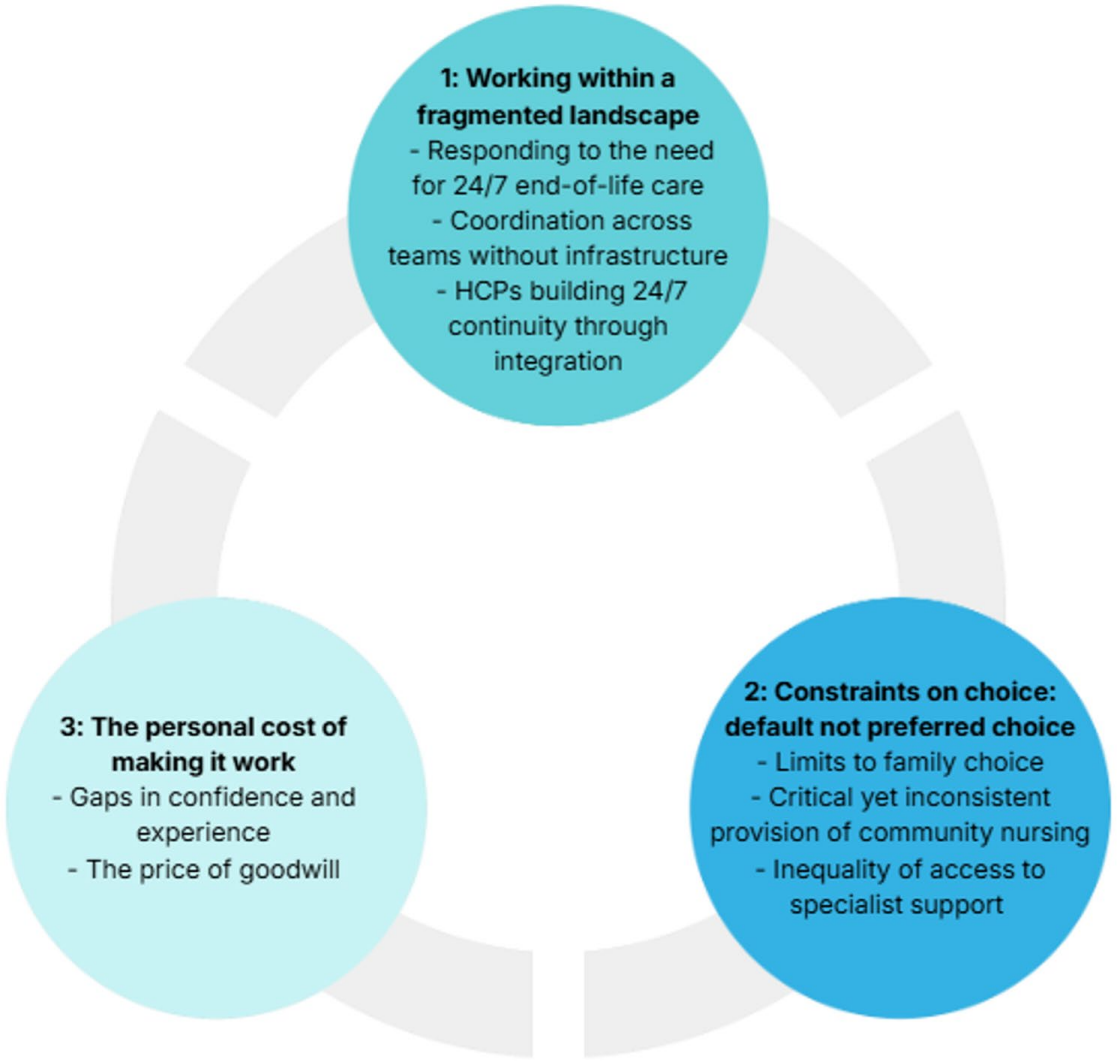
Themes and subthemes

Three themes with sub-themes were developed (see supplement 2 for definitions). These are presented separately, however there is also intersectionality. Working within a fragmented landscape (theme 1) directly shapes the constraints on choice: default not preferred choice? (theme 2) which in turn impacts on health professionals who pay the personal cost of making it work (theme 3). Figure 1 illustrates this interconnectedness.

### Working within a fragmented landscape

HCPs were constantly navigating fragmented services and inconsistent commissioning to deliver 24/7 care to families with a child at the end-of-life, often requiring them to ‘patch together’ bespoke solutions.

#### Responding to the need for 24/7 end-of-life care

There was stark variation in how professionals from teams in different areas and services were able to respond when a child and their family needed around the clock end-of-life care. A select few teams, the larger hospices and a tertiary oncology outreach service, had existing systems and staff in place to provide seamless 24/7 cover. The same team of professionals who had been supporting a family through their care journey continued to provide support no matter the time or day as a child reached the end-of-life. They were able to continue their relationships with families and knew the child’s medical background and the family context. This established trust and continuity made supporting them around the clock easier.

*It’s about building that relationship*,* and I think it is really helpful actually*,* when they have got that relationship and they know they can phone and run it past somebody*,* even if it is 3:00am in the morning*,* and then move forward. (FG01-01 Hospice SPPC Consultant)*

Services operated very different models; for example, one hospice provided a 24-hour access to consultant-led advice line (for families and professionals) with back up face-to-face support either from the hospice care team or local community children’s nursing (CCN) teams. This service was available to the entire hospice caseload, not just those who are receiving end-of-life care, meaning there was no transition or change in services for families at that difficult time. The oncology team had a standing rota of clinical nurse specialists who are continually on call across their area. When a child reached the end-of-life phase, support stepped up and their family were provided with the details of the on-call rota so they could access teams support as and when needed.

*We do all key work a geographic patch*,* but with the on call out-of-hours*,* we cover everybody*,* so we have our own caseloads*,* but we cover everybody on call. (FG05-01 Oncology CNS)*

In parts of the region where an existing mechanism for 24/7 care was not in place, and a child reached the end of their life, HCPs responded on a case-by-case basis to pull a team together to support them. Local variations in funding, provision and coverage of children’s community nursing, palliative care teams, and hospice support meant that each family needed a bespoke solution.

*We’re kind of having to mishmash things together to try and make it work for the family and give them the experience that they should deserve. (FG08-01 Community Children’s Nurse)*

In a handful of localities there was a commissioning structure to fund temporary services, this relied on teams knowing local ICB Commissioners and putting the case about a specific child and their needs. However, funding was not guaranteed, making planning and discussions with parents difficult.

*We’ve got a really good relationship with the commissioners so*,* you know*,* we’ve got the mobile numbers*,* and we might ring them and say*,* we’ve got a child*,* we think they’re end of life*,* the family’s desperate to get home and they are brilliant. They’ll pull out all the stops*,* they’ll say*,* fine*,* we can secure the funding*,* that’s no problem for however long*,* you know*,* I mean*,* not for however long*,* I think we’ll provide it for the next three months. (FG04-02 Palliative Care Service Nurse)*

In many areas, extra funding was not accessible, and it was a case of relying on staff ‘stepping-up’ and rotas being reorganised on a temporary basis. Issues of staff shortages, variations in skill levels and confidence, coupled with uncertainty around how long support was needed made planning extremely hard.

*It’s just so variable isn’t it and we do have teams around the region who will step up and do an ad-hoc*,* goodwill*,* 24/7 service and we have those that absolutely can’t and you know we’ve got community teams that are staffed by band 5 nurses versus community teams who have got band 7 complex care nurses*,* it’s really variable (FG03-05 Palliative Care CNS)*.

#### Coordination across teams without infrastructure

It was evident that coordinating 24/7 support for families, particularly when it involves professionals from across services, took time. Professionals described how the basic practicalities such as ensuring families knew who, when and how to contact them were not straightforward. Each local area had a different solution. It was highlighted how there was rarely a single point of contact for families to ring out-of-hours, and several described having to provide their mobile numbers to families. This led to HCPs feeling beholden to other families and having difficulties putting boundaries in place.

*But then I’ve also gave them my works mobile number but then keeping that on overnight people don’t understand that it’s 10 o’clock at night and I’m not going to answer their phone*,* so I have other people ringing me and I’m thinking*,* “Oh*,* do I need to answer the phone to them*,*” and that stresses me out a bit but I was just thinking like how do you work it as in how they contact you? (FG08a-01 Community Children’s Nurse)*

Sharing an ‘on call’ phone worked for some teams; however, handovers became logistically complicated if it was a wide geographic area. Other solutions included using the local children’s ward or a respite centre as a first port of call for families. One CCN team were working closely with a hospice, which was not local, but was staffed overnight. The hospice care team provided an out-of-hours ‘triage’ service and called the local CCN team if a family needed someone to go out to them.

A clear point of frustration for many professionals was the lack of integration of computer systems and patient records. This meant they had to rely on team members proactively updating, emailing or phoning colleagues to make sure everyone is on the same page and up to date.

*So*,* I think part of the problem is using different systems in different settings. So*,* an out-of-hours service uses [IT system X] or the in-house service uses [IT system Y] most of the time and they do communicate a bit*,* but you can’t see advanced care plans from one to the other until it takes somebody to actually say*,* please send this information*,* which doesn’t happen all the time. (FG08-02* GP)

Ensuring that care plans are in place and shared appropriately was seen as key, so that everyone supporting a family understand the situation, the arrangements in place and the steps to take if something changed. Several mentioned the specific need for ambulance services to be informed so that usual protocols do not automatically kick in.

*And I think care plans are maybe a bit more important for some of our children because we don’t have any out-of-hours provision*,* so if they’re coming into contact with other professionals out-of-hours it’s professionals that don’t know them*,* so that’s all they’ve got to rely on*,* the advanced care plan. (FG03-04 Palliative Care Nurse Specialist)*

To counter the lack of infrastructure and to ensure continuity of approach, the importance of proactive communication including a good ‘handover’ between teams was emphasised.

#### HCPs Building 24/7continuity through integration

Professionals recognised they needed to work together across services and boundaries to find sustainable solutions that work within the specific context of each area to provide 24/7 support for families.

*We are looking at ways that we could maybe work out some inter-organisational agreement to do that*,* but I think that’s very much early stages of discussion and even so we’re still recognising that we’re a small team and we need to be sustainable because actually if we all fall apart then the region has nothing. (FG03-03 Specialist Palliative Care Service Consultant)*

Physical integration of teams, in terms of co-location, meant that HCPs knew each other well and were able communicate easily and informally, leading to increased efficiency and improved care experiences for families.

There were also examples of teams integrating to provide broader coverage, including one larger hospice providing out-of-hours phone support to an area covered by a smaller hospice, with a rota of local CCNs on call if needed. In another area several teams were working together to put a pre-emptive on-call rota in place so it can be instigated quickly.

*We are working very closely with our other ICBs and our other Palliative Care Community Teams*,* so especially with the Specialist Nursing Teams. So*,* what we are trying to do is put sort of an on-call rota in place so if any child was to become particularly unwell and wished to pass away at home*,* we don’t have to manage it just as one team and everybody struggle. We can manage it collectively as sort of a group of teams. So*,* what we are trying to do is get some form of agreement in place*,* so we all know what we are doing when we get to that point. (FG01-03 Hospice Head of Children’s & Wellbeing Services)*

Some specialist palliative care consultants had dual roles across two organisations, generally working in an NHS specialist palliative care team and a hospice. This facilitated coordination and communication between these teams, as well as reducing IT barriers as they had access to all the systems.

*I think the two hats approach is absolutely brilliant*,* because. these are the same children*,* they are the children at home*,* they are the children in hospitals*,* and these children with complex needs will be going into hospital and they will at times want to be staying out of hospital…. So*,* I have access to all their computer records*,* which is hugely beneficial for the patients and for us. (FG01-01 Specialist Palliative Care Consultant)*

Another approach was to build local palliative care capacity and skills. Regionally there were several examples of initiatives including: embedding palliative care nurses in children’s community nursing teams, a specialist palliative consultant holding clinics in local hospitals with their generalist colleagues and the integration of a hospice nurse with an oncology multi-disciplinary team.

*There’s not going to be hundreds of specialist palliative care consultants…. So*,* they’re not going to be enough of those kinds of people*,* so you need to be able to skill up other more generic paediatricians to be able to help deliver with other personnel*,* nurses and so on. (FG07-03 Consultant Paediatrician)*

### Constraints on choice: default not preferred choice?

Professionals strived to offer and then provide families with care in their preferred place; however, the choices available were very much constrained by the availability of local services, in particular Community Children’s Nursing and Specialist Paediatric Palliative Care.

#### Limits to family choice

Professionals emphasised the importance of offering families *informed* choice about where their child received end-of-life care. They ensured parents had an honest picture of the options available and what support could be delivered and then discussed whether this met their needs. HCPs acknowledged that parent choices varied considerably and are shaped by many things including the child’s symptoms, previous experiences of care, their relationships with HCPs, and personal preferences of the role they as parents want to play.

*And so*,* whilst we encourage parent choice that needs to be an informed choice and that informed choice is very dependent on (1) the actual diagnosis*,* (2) the ability of the parent but also (3) what’s available and so on. (FG03-02 Specialist Palliative Care Consultant)*

Some families wanted autonomy and to be upskilled, others wanted continual support and care, so it was clear that a family centred approach is required.

*We teach them to give buccal medications*,* or we teach them to give PRNs through the subcut device. So*,* some families do that amazingly well and that’s what they want to do because they don’t want people in the house*,* and they’ve got really good support*,* and they’ve got grandma*,* and they’ve got mum and dad. But then we’ve got other families who are just thinking*,* I absolutely can’t do that for my child and please don’t make me do that. (FG04-02 Palliative Care Service Nurse)*

HCPs understood often families in the region wanted to be at home with 24/7 seamless support from a team they knew, however the reality was for many, the services were not available to support that choice, and professionals had the hard job of explaining this to families.

*So*,* it’s so hard to promise and the only thing that’s guaranteed is if you stay in hospital*,* you will get a nurse. If you go to a hospice*,* you will have people around. We can’t guarantee any more than that. It does feel like home isn’t an option because we don’t have 24-hour carers (FG04-02 Palliative Care Service Nurse)*.

Parts of the region were not served at all by children’s hospices, or there were recent reductions in the services offered, including not being able to provide end-of-life care, further restricting the choice.

*We do support families in their preferred place of death*,* but when the option is home or hospital*,* your preferred place of death is somewhat limited (FG05-03 Oncology Team Consultant)*.

Professionals described how some families, whose child had spent a long time in hospital and for whom continuity was important, chose for their child to die in that familiar ward setting. While this was described by several professionals as a ‘compliment’ and a reflection of the support they had given these families, it was also recognised that some of these families made this choice by default as they didn’t feel safe going home.

*A lot of our families spend so much time [on the ward] it becomes*,* in a way*,* their safest place…. they know they’ve got the support*,* they know they’ve got the familiar faces as opposed to it being a purely at home this is going to be quite scary and quite difficult (FG04-01 Physiotherapist)*.

HCPs described personal and professional satisfaction when they were able to support families to achieve their preferred place of care and death.

*Mum said “We can’t thank you enough for allowing her to be at home and to die at home with her family” and every time I go*,* I get goosebumps arriving at the house to see the family because I think hopefully they have had the best experience in what was the worst situation to be in*,* and that’s why we do it*,* isn’t it? And yeah*,* the challenges are there*,* but I would take the challenges and provide that service every day*,* you know. I think it is a privilege. (FG05-01 Oncology Team CNS)*

However, when faced with service gaps and an inability to facilitate child and parent choice due to inequality, they felt the impact of this moral distress profoundly.

*I’ve had a young lad*,* whom I will never forget*,* who was non-verbal because he had lost his ability to speak but his cognition hadn’t declined so he was still signing and he’d been in a hospice for 75 days on a syringe driver and he signed*,* “Home*,*” to me and I couldn’t send him home because there was no support available for home and he died in the hospice. I’ll never forget that you know. (FG3a-07 Specialist Paediatric Palliative Care Consultant)*

#### Critical yet inconsistent provision of community nursing

A recurring theme was how the current fragility of resourcing of children’s community nursing (CCN) was a major constraint to offering families real choice in preferred place of care. CCNs operate at local level and often have well established long-term relationships with families. There were examples from a few teams who were well resourced and were able to successfully support parents with their choices.

*I think it’s worth saying that in the whole time I’ve been in our service we’ve never not managed to support a child in the community who’s been dying at home. (FG08-09 Children’s Community Nurse)*

However, in other areas, staff shortages and funding gaps meant that many CCN services had reduced their hours, and in some areas, there was no provision at all. This reduced capacity has also had direct implications for other teams. The paediatric oncology teams all mentioned that their workloads were increasing significantly because of these changes.

*So*,* we meet regularly with our CCN teams across all the patches… and they will then give their offer of what they can do for the end-of-life phase. A lot of them will only be able to support when the syringe driver goes on*,* which is brilliant*,* because it’s like 24 h and then you’ve got to go change it. … It’s the weekends that we struggle with*,* the cover. They’re not commissioned for that*,* so then we would pick that up*,* yeah. (FG05-02 Oncology Team Nurse)*

The local variation in commissioned provision and therefore teams’ capacity to provide end-of-life care meant some community nurses were not getting relevant training or hands-on experience, which in turn limited their ability to provide appropriate care.

*Most CCN’s cannot extend their hours or put ad hoc rotas up but more importantly because they haven’t been doing that*,* they don’t have the skills or the confidence (FG03a-07 Specialist Paediatric Palliative Care Service Consultant)*.

HCPs supporting families understood the local variation and context for individual CCN teams and were very aware of the ability of specific teams to provide 24/7 care or not. They liaised with the networks of local teams to negotiate the level of support they could offer a family and at what point, and this was a significant factor when planning where care for a child could be provided.

*We’re recognising some of the community nursing teams are very*,* very confident and really good at end-of-life care*,* and some of them are floundering or just stretched beyond their limits and so what we need to plan around is quite often based on our knowledge of what’s available locally as well. (FG03-03 Specialist Palliative Care Service Consultant)*

Professionals recognised the current system was unstainable and new creative models of delivery need to be considered. Suggestions included the use of a flexible team of expert community nurses covering the whole region or using hospice nursing teams as rapid response to be in families’ homes.

#### Inequality of access to specialist support

Supporting a child at the end-of-life is complex and to confidently provide this care, particularly at home, HCPs, particularly CCNs, wanted the security of knowing they could access advice and input from a specialist palliative care team, day and night. Having the backup of 24/7 specialist expertise reduced HCP stress and isolation.

*Without them [Hospice] we literally would not cope*,* we’re not the experts in end of life*,* we’re absolutely not*,* yes*,* we’ll do it with support*,* but if we didn’t have that*,* we would be very lost so yeah*,* I think we’re just very lucky. …they’re always on the end of the phone*,* you can literally speak to anybody*,* so you’re never on your own*,* you don’t feel isolated. (FG08a-03 Community Children’s Nurse)*

This was echoed by on-call Specialist Palliative Care team consultants who reflected that most of the overnight calls they got were from other HCPs supporting a child at the end of life. However, within the region there was only one service, based in a hospice, that had a full rota of specialist palliative care consultants providing 24/7 cover to their caseload and to other HCPs. In the other areas specialist palliative care consultant posts were only funded part-time and usually weekdays only, and palliative care nursing teams were also generally only commissioned for office hours.

Another barrier was that in some situations HCPs could only access 24/7 specialist advice about a patient if they were under the care of the hospice. This meant that they found themselves pushing for a referral even when the families were reluctant engage with the hospice.

*His dad kept saying*,* “We don’t need a referral. We know the hospice is there*,* we’re not there yet” eventually I said to him*,* “I would really appreciate it if you would let me refer your son to a hospice because I know at the moment*,* you’re coping really well… but sometimes I need that support. I need that phone a friend to go do you know what*,* I’m really worried about this patient*,* and this has happened and that’s happened and have you got any ideas” (FG04-02 Palliative Care Service Nurse)*.

Regional inequality of specialist palliative care provision was personally distressing for some HCPs, not just for themselves, but also for the families who were not able to benefit from access to a well-supported care team.

*There are hundreds of children regionally that don’t have access to a specialist service at the moment and especially with our lack of hospice.*,* there are lots of community teams that haven’t had the support that they need from a hospice or a specialist team in the past year or two which has been really sad and families that haven’t had access to that support. (FG03-04 Palliative Care Nurse Specialist)*

Professionals reported feeling discomfort about disparity by diagnosis. In all areas of the region families of children with a cancer diagnosis at the end-of-live were supported at home (if that’s what they wanted) by round the clock care from the paediatric oncology outreach nursing teams. For HCPs caring for children with other diagnoses this felt unfair.

*So usually if you’ve got cancer*,* you get a great service*,* amazing service*,* no complaints at all but there’s obviously this greater cohort of children with sort of neuro disabling conditions or genetic conditions are underserved. and it’s a question about why there’s such a mismatch between children with cancer*,* which is a relatively rare diagnosis in children compared to this larger cohort of children with palliative care needs. (FG07-03 Consultant Paediatrician)*

Several members of Specialist Palliative Care Teams, reflected that they could do a better job at ensuring that other HCPs across the region know what support is available to them. Professionals often did not know they could call for advice, or to consult about a patient, and were particularly cautious about ringing in the middle of the night.

*The more junior staff would sometimes say to this parent*,* “I can’t wake that doctor overnight. I can’t ring the hospice*,* I can’t ring them*,*” and the parent was repeatedly having to say*,* “No*,* you can*,* if I was at home*,* I would just be picking up the phone.” So*,* there’s a staff understanding of the systems (FG04-03 Consultant Paediatrician)*.

### The personal cost of making it work

Professionals described the emotional and physical burdens they caried as they stretched themselves to provide end-of-life care, often having to sacrifice their personal wellbeing and in doing so, inadvertently sustaining a broken system.

### Gaps in confidence and experience

Due to the relatively low number of child deaths a year, providing round the clock end-of-life care for children is infrequent, and so some generalist professionals, both in the community and in hospitals, perceived themselves to be lacking experience.

*I think the confidence and the competence of clinical staff in communities and on some wards as well*,* there are huge gaps. Coming from an adult world where in palliative care is much more bread and butter and I think more people get to a basic level of palliative care confidence very quickly working in both nursing and medicine. Coming to paediatrics there are huge gaps in people’s experience and confidence around end-of-life care*,* so we are putting more and more emphasis on training in our region to try and upskill people and given them that confidence. (FG03-03 Specialist Palliative Care Service Consultant)*

It was recognised that delivering 24/7 symptom management for a child at the end of life is medically complex and inexperience can mean that professionals were cautious, or in some case reluctant to give necessary medications.

*We bang our head against the wall sometimes and people are saying*,* oh no*,* we can’t give Morphine*,* it’ll suppress the respiratory drive and then that creates a culture of no*,* we’re not doing symptom management because we’re frightened of it. (FG04-02 Palliative Care Service Nurse)*

Limited confidence, experience and training impacted resource demand. For example, managing syringe drivers, used for delivering pain relief, often required daily attendance and urgent action if they malfunctioned. While some teams were confident managing them alone, others required two staff members, placing additional pressure on staffing and rota.

*If we said an end-of-life patient is going to ring and if there’s anything wrong with the syringe driver they just panic instantly*,* it’s not something that they do (FG08a-01Community Children’s Nurse*).

HCPs described a shift in focus during the end-of-life phase, emphasising quality of life, time with family, and memory-making, over active treatment. They acknowledged this transition can be challenging, especially for less experienced staff, highlighting the need to ensure appropriate emotional and psychological support is in place when planning and anticipating a child’s death.”

*Because there’s quite a lot of new members to the team*,* young members to the team*,* who this is the first death that they’ve experienced*,* so we are seeing that we need to be quite mindful of that and how we’re actually supporting the team with that. (FG03-02 Specialist Palliative Care Consultant)*

Professionals in some settings rarely provided end-of-life care, and so it was difficult to maintain the relevant skill levels and competencies.

#### The price of goodwill

The final sub-theme recognises the personal toll that service gaps take on professionals. In most local areas professionals had to react, adapt and ‘step up’ to provide families with a dying child the care they need. Many described working extra hours and providing on-call cover out of goodwill as there was simply no other option.

*We do goodwill*,* so we’ve been known to go out actually one winter*,* full of snow*,* no-one could get to this family*,* needed syringe driver on a weekend so [Hospice] team were involved*,* I think the Macmillan team were involved too but no-one could get to this family who were quite rural*,* so [CCN] bless her*,* got the train and then walked about three miles or something. And that’s on a weekend and then we’ll just claim our time back. (FG08a-03 Community Children’s Nurse)*

Staff shortages in many teams meant providing round the clock end-of-life care had significant strain on the entire team, especially those were small, or included part-time staff.

*It’s really difficult because we’re not just a palliative care team*,* so it is*,* for want of a better word*,* it is a real headache to try and put a palliative care rota together. It’s like doing a constant Jenga and then you’ve got sickness and annual leave to think about as well. We always have two people… so again that impacts on the wider team and what they can do the next day. (FG08a-04 Manager*,* Children’s Community Nursing)*

Supporting a family overnight is tiring and comes with the burden of always being available. However, the administrative systems were often not in place to support ad hoc ‘stepping’ up, with rotas and pay mechanisms too rigid to be able to ensure that HCPs who are working extra hours or overnight were compensated or even given time off to rest.

*You’re up constantly every hour*,* you’re looking at your phone*,* you’re making sure they’re not ringing you*,* you’re worried that you’re going to miss that call and then you’re in work the next day - our caseload doesn’t change. Then it ends up that you’re working seven days a week*,* 24/7*,* it does become really difficult*,* and again it’s just a goodwill gesture*,* we don’t get any time back. It can be really draining*,* it can be really tiring*,* but obviously we do what we have to do for the patients. (FG08a-01Children’s Community Nurse)*

A key difficulty is planning and then sustaining a ‘temporary’ rota. For children, the end-of-life phase can be unpredictable, and it was often unclear how long 24/7 support would be required. This meant that once professionals ‘flexed up’ on a temporary basis it was hard to draw back from that without letting a family down.

*I think one of the other things we have found difficult is that we can put in an ad-hoc rota*,* but some children die very quickly*,* and some children take quite a long time*,* and it is difficult to know how long to plan for. I think in the last couple of years we have had a couple of situations where we have ended up sort of doing a one in two rota for a couple of months even*,* which has just been punishing. There was never a point that felt suitable to pull back*,* because they looked like they would be dying for quite a long time. So*,* I think it is just that unpredictability*,* (FG01-08 Consultant Palliative Medicine)*

This has real implications for the personal wellbeing for professionals, many of whom described how current system constraints meant that care was being delivered at a personal sacrifice to themselves.

*I don’t even think we’re running on goodwill anymore. I think we’re running on the fumes of goodwill because everybody’s so knackered and with the recruitment and retention issues*,* we don’t have that pool. (FG04-02 Palliative Care Service Nurse)*

*The cost is to the individuals. They’re absolutely exhausted*,* they’re absolutely wiped out but will keep on doing and keep on doing. (FG05-02 Oncology Care Team Lead Nurse)*

But for many the personal cost is worth it, as universally HCPs wanted to provide the care that families need, and so when the systems and resources were in place to ensure this was delivered, then professionals felt privileged to be involved.

*It is a privilege*,* and it is very rewarding*,* and actually to empower those families to have some control at the worst possible time in their life*,* it is just – you can’t explain it*,* can you? And to be able to fulfil that for them is what drives you to keep going. And I do think you have to be a certain type of person to be able to keep going and have the resilience. (FG05-01 Oncology Care Team CNS)*

It was acknowledged by a few professionals that one inadvertent consequence of people working extra hours, filling critical gaps and supporting families out of goodwill, is that the true extent of the system problems is hidden and there is therefore less pressure for reform.

*You sometimes need to create a crisis to get the recognition*,*” and I said*,* “It’s very difficult for you if you are the person dealing with the patient to create a crisis because you don’t want the patient to suffer because you’re trying to make a national crisis.” (FG07-06 Community Paediatrician)*.

*I think so many people go above and beyond and that’s why this hasn’t ever been addressed or services really thought of. (FG03a-08 Specialist Paediatric Palliative Care Service Nurse)*

## Discussion

This study found that healthcare professionals in the region were constantly navigating fragmented services and inconsistent commissioning to deliver 24/7 care to families with a child at the end of life. They strived to offer families care in their preferred place; however, the reality was that choice was constrained by the availability of local services, in particular Community Children’s Nursing and Specialist Palliative Care. Professionals stretched themselves to provide around the clock care, often having to sacrifice their personal wellbeing and in doing so, inadvertently sustaining a broken system.

HCPs recognised that when teams were able to provide continuity and a seamless transition to 24/7 end-of-life care, a trusted relationship was established which empowered parents and enabled children to remain at home as long as they wanted, this reflects other research, including a parallel workstream to this study focusing on parental experiences of 24/7 care [19, 24, 25]. HCPs felt a keen sense of professional satisfaction and privilege when were able to support families around the clock, echoing the experiences of professionals providing end-of-life care in acute settings [26]. 

Professionals described the impact of service gaps, and the extra work entailed ‘patching together’ bespoke solutions, including securing funding, stretching rotas and working out of goodwill, amidst a lack of integrated IT, and rigid governance and administrative infrastructures. Improved functional integration such as shared electronic patient records [27] and flexible payroll systems that allow staff to be compensated appropriately are needed. The proposed push to digital in the NHS Long Term plan [28], including single patient records and My Carer app for parents should facilitate cross team co-ordination.

The key role of local CCNs to support 24/7 end-of-life care was strongly emphasised, as was the impact of the significant variation in this provision. Current workforce levels are a long way from Royal College of Nursing recommended safe staffing levels [8], and research shows nearly half of CCN teams refusing referrals due capacity issues [29]. Reduced opportunity for CCNs to be involved in the provision of end-of-life care meant that some teams have become deskilled and now lack the confidence to ‘step up’ when required. Inconsistent commissioning and shortages of specialist paediatric palliative care services stressed by the Commission for Palliative and End-of-life Care [18], were also apparent in this study, along with wide variation in the availability and provision of charity funded hospice services. A survey of UK hospital and hospice based palliative care teams found only a third met National Institute for Health and Care Excellence minimum staffing criteria [30]. 

Professionals described the moral distress they felt when faced with inequity and an inability to provide children and their families with high quality 24/7 end-of-life care in their place of choice, an impact observed in other settings [26, 31, 32]. Previous research has also highlighted the emotional toll on professionals of providing end-of-life care [26, 33, 34], this study goes further, demonstrating that the emotional and physical price of professionals on the ground ‘stepping up’ to fill 24/7 service gaps, often out of goodwill, is not only unsustainable but was also seen to inadvertently reinforce the current broken system.

The Commission on Palliative and End-of-life Care recommends that access to 24/7 specialist palliative care should be mandated and funded, working in coordination with general care in every area [18]. Given the local variations in context, as shown in this paper, there is no blueprint for how services should be organised to provide this care when required [9]. It is important to draw on the pockets of innovative 24/7 service design that are in place across the UK and internationally [24, 25, 35]. Professionals acknowledged the difficulties of planning and providing 24/7 end-of-life care at a local level to a geographically dispersed small number of children, with a diverse range of complex conditions [16]. This study shows there is a need to create economies of scale, collaborate and integrate, and to build on locally driven initiatives that were emerging including ICBs working jointly or across borders, capacity building and knowledge transfer.

The study has demonstrated the need for specialist service integration, potentially across ICB boundaries, and coordination with other teams including local CCNs, at a scale that is sustainable. This may be at odds with the central ambition of the NHS Long Term Plan to deliver services, including palliative care, at a neighbourhood level [28] ICBs will continue to miss national standards for 24/7 end-of-life care for children [5, 8] unless they work jointly to commission and deliver specialist palliative care services in partnership with local CCN teams and children’s hospices.

A strength of this study is that it explores the perspectives of professionals in a large English region with 4 ICBs, and a wide range of models of care. A targeted recruitment strategy ensured that the sample had good representation from community, hospital and hospice services and settings across the region, and participants had varied levels of experience of delivering end-of-life care. Organising data collection so that professionals in similar roles and settings were brought together was logistically complicated, but this then allowed for rich discussion of how approaches differed by location and provided an opportunity to exchange ideas. One group of professionals not well represented in the sample was GPs. There is further work to be done to establish the optimal geographic areas at which specialist services should be delivered.

## Conclusion

Stepping up to support families with 24/7 end-of-life care for their child, has resulted in an unsustainable physical and emotional toll on professionals. The impact of delivering care in an inequitable system is causing significant moral distress, and there is a growing realisation that their goodwill is masking current systemic shortcomings. ICBs must work jointly to find economies of scale to establish equitable and sustainable models of delivery that meet national standards and to ensure all children have access to high quality 24/7 end-of-life care.

## Supplementary Information

Supplementary Material 1

Supplementary Material 2

## Data Availability

The dataset generated and analysed during the current study is not publicly available due to ethical considerations (to ensure data confidentiality and protect the anonymity of the research participants) but is available from the corresponding author on reasonable request.

## References

[1] Cooper J. End of life care: strengthening choice. An inquiry report by the All-Party Parliamentary Group (APPG) for Children Who Need Palliative Care England. 2018. https://www.togetherforshortlives.org.uk/app/uploads/2018/10/Pol_Res_181019_APPG_Children_Who_Need_Palliative_Care_inquiry_report.pdf.

[2] Benini F, Papadatou D, Bernadá M, et al. International standards for pediatric palliative care: from IMPaCCT to GO-PPaCS. J Pain Symptom Manage. 2022;63:e529–43. 10.1016/j.jpainsymman.2021.12.031. 35031506 10.1016/j.jpainsymman.2021.12.031

[3] Fields D, Fraser LK, Taylor J, Hackett J. What does “good” palliative care look like for children and young people? A qualitative study of parents’ experiences and perspectives. Palliat Med. 2023;37(3):355–71. 10.1177/02692163231154300. 36825577 10.1177/02692163231154300 PMC10021114

[4] NHS England. Service specifications for palliative and end of life care: children and young people. London: NHS England; 2023.

[5] National Institute for Health and Care Excellence (NICE). End of life care for infants, children and young people; quality standards. London: National Institute for Heath and Care Excellence; 2017. London. https://www.nice.org.uk/guidance/qs160.

[6] European Association for Palliative Care (EAPC) Children and Young People’s Reference Group. European Charter on Palliative Care for Children & Young People. EAPC; 2023.

[7] National Palliative and End of Life Care Partnership. Ambitions for palliative and end of life care: A national framework for local action 2021–2026. London: NHS England; 2021.

[8] Together for Short Lives. Built to Last? The state of children’s palliative care in 2025; 2025. https://www.togetherforshortlives.org.uk/app/uploads/2025/05/Built-to-Last-The-state-of-childrens-palliative-care-in-2025.pdf.

[9] Malcolm C, Knighting K, Taylor C. Home-based end of life care for children and their families - a systematic scoping review and narrative synthesis. J Pediatr Nurs. 2020;55:126–33. 10.1016/j.pedn.2020.07.018. 32949852 10.1016/j.pedn.2020.07.018

[10] The National Child Mortality Database (NCMD). Child Death Review Data Release: Year ending 31 March 2024,, https://www.ncmd.info/publications/child-death-review-data-release-2024/ (2024, accessed 26/02/24).

[11] Hammer NM, Bidstrup PE, Brok J, et al. Home-based specialized pediatric palliative care: a systematic review and meta-analysis. J Pain Symptom Manage. 2023;65:e353–68. 10.1016/j.jpainsymman.2022.12.139. 36621694 10.1016/j.jpainsymman.2022.12.139

[12] Winger A, Kvarme LG, Løyland B, et al. Family experiences with palliative care for children at home: a systematic literature review. BMC Palliat Care. 2020;19:165. 10.1186/s12904-020-00672-4. 33099303 10.1186/s12904-020-00672-4 PMC7585197

[13] Widdas D, McNamara K, Edwards F. A core pathway for children with life-limiting and life-threatening conditions. Bristol: Together for Short Lives; 2013.

[14] Noyes J, Edwards RT, Hastings RP, et al. Evidence-based planning and costing palliative care services for children: novel multi-method epidemiological and economic exemplar. BMC Palliat Care. 2013;12:18. 10.1186/1472-684X-12-18. 23617814 10.1186/1472-684X-12-18 PMC3651264

[15] Hain R, Heckford E, McCulloch R. Paediatric palliative medicine in the UK: past, present, future. Arch Dis Child. 2012;97:381. 10.1136/archdischild-2011-300432. 22039176 10.1136/archdischild-2011-300432

[16] Papworth A, Hackett J, Beresford B, et al. Regional perspectives on the coordination and delivery of paediatric end-of-life care in the UK: a qualitative study. BMC Palliat Care. 2023;22:117. 10.1186/s12904-023-01238-w. 37587514 10.1186/s12904-023-01238-w PMC10428585

[17] Gheera M, Plaskitt H. Funding for Children’s Hospices. [House of Commons Library Debate Pack CDP-2024-0140]. London House of Commons Library; 2024.

[18] Finlay I, Richards M, Maskell R, et al. Palliative care and End-of-Life care: opportunities for England (Volume 1). The Commission on Palliative and End-of-Life Care; 2025.

[19] Barrett L, Fraser L, Ziegler L. et al. “We’re teetering on unsteady ground” parents’ experiences of accessing 24/7 paediatric end-of-life care: a qualitative study. BMC Palliative Care. 2025;24:285. 10.1186/s12904-025-01927-8. 10.1186/s12904-025-01927-8 PMC12613421 41225466

[20] Braun V, Clarke V. Using thematic analysis in psychology. Qual Res Psychol. 2006;3:77–101.

[21] Braun V, Clarke V. Thematic Analysis: A Practical Guide. London: Sage; 2022.

[22] Tong A, Sainsbury P, Craig J. Consolidated criteria for reporting qualitative research (COREQ): a 32-item checklist for interviews and focus groups. Int J Qual Health Care. 2007;19:349–57. 17872937 10.1093/intqhc/mzm042

[23] Gale NK, Heath G, Cameron E, et al. Using the framework method for the analysis of qualitative data in multi-disciplinary health research. BMC Med Res Methodol. 2013;13:117. 10.1186/1471-2288-13-117. 24047204 10.1186/1471-2288-13-117 PMC3848812

[24] Malcolm C, Knighting K. A realist evaluation of a home-based end of life care service for children and families: what works, for whom, how, in what circumstances and why? BMC Palliat Care. 2022;21:31. 10.1186/s12904-022-00921-8. 35255888 10.1186/s12904-022-00921-8 PMC8902768

[25] Hammer NM, Hansson H, Pedersen LH, et al. Intersectoral collaboration in home-based end-of-life pediatric cancer care: a qualitative multiple-case study integrating families’ and professionals’ experiences. Palliat Med. 2022;37:149–62. 10.1177/02692163221135350. 36397271 10.1177/02692163221135350

[26] McLorie EV, Hackett J, Barrett L, et al. Healthcare professionals’ perspectives of providing end-of-life care for infants, children and young people in acute settings: A multi-site qualitative study. Palliat Med. 2025;02692163251320204. 10.1177/02692163251320204. 10.1177/02692163251320204 PMC11977802 39995206

[27] Lewis R, Rosen R, Goodwin N, Dixon J. Where next for integrated care organisations in the english NHS? London: Nuffield Trust and King’s Fund; 2010.

[28] Department of Health and Social Care: 10 year health plan for England: fit for the future. London: Department of Health and Social Care; 2025.

[29] The Queen's Nursing Institute. Community Children’s Nursing in the 21st Century: Developing and Strengthening CCN Services. London: The Queen’s Nursing Institute; 2019.

[30] Bedendo A, Hinde S, Beresford B, et al. Consultant-led UK paediatric palliative care services: professional configuration, services, funding. BMJ Supportive Palliat Care. 2024;14(e554). 10.1136/spcare-2023-004172. 10.1136/spcare-2023-004172 37558392

[31] Tan L, Sheri S, Goh YY, et al. Experiences of healthcare professionals providing palliative care in home settings - a scoping review. BMC Palliat Care. 2025;24:83. 10.1186/s12904-025-01728-z. 40155860 10.1186/s12904-025-01728-z PMC11951797

[32] British Medical AssociationMoral distress and moral injury – recognising and tackling it for UK Doctors. London: British Medical Association; 2021.

[33] Neilson S, Kai J, MacArthur C, Greenfield S. Exploring the experiences of community-based children’s nurses providing palliative care. Paediatr Nurs. 2010;22:31. 20426356 10.7748/paed2010.04.22.3.31.c7640

[34] Reid F. Grief and the experiences of nurses providing palliative care to children and young people at home. Nurs Child Young People. 2013;25(9):31–36. 10.7748/ncyp2013.11.25.9.31.e366. 10.7748/ncyp2013.11.25.9.31.e366 24200187

[35] East of England children’. s palliative care service launched. https://www.cuh.nhs.uk/news/east-of-england-childrens-palliative-care-service-launched/ (2022, July 20).

