# Supplementary material for: “We are running on the fumes of goodwill” Professionals’ experiences of delivering 24/7 end-of-life care to children and their families: a qualitative study
Source: BMC Palliat Care. 2025 Dec 3;25:9. doi: 10.1186/s12904-025-01958-1 (PMC12781730; doi:10.1186/s12904-025-01958-1)
Supplement: Supplementary file 1 — Supplementary Material 1 [file 12904_2025_1958_MOESM1_ESM.docx]

**“We are running on the fumes of goodwill.” Professionals’ experiences of delivering 24/7 end-of-life care to children and their families: a qualitative study**

Supplementary file 1: Healthcare Professionals Focus Group - Summary Topic Guide

1. **Preliminaries**
2. **Introductions**
   1. **Quick background introductions:**
   2. **Brief description of services provided**
3. **Supporting families at home**
   1. **Planning and decision making**
   2. **Contacting the hospice**
   3. **Staff rotas**
   4. **Liaising with other professionals**
   5. **For hospices that are not currently providing EoL care**
   6. **End-of-life OOH care**
4. **Supporting colleagues across the region**
5. **Gaps, needs and suggested improvements**
6. **Close**
