# Supplementary material for: “We are running on the fumes of goodwill” Professionals’ experiences of delivering 24/7 end-of-life care to children and their families: a qualitative study
Source: BMC Palliat Care. 2025 Dec 3;25:9. doi: 10.1186/s12904-025-01958-1 (PMC12781730; doi:10.1186/s12904-025-01958-1)
Supplement: Supplementary file 2 — Supplementary Material 2 [file 12904_2025_1958_MOESM2_ESM.docx]

**“We are running on the fumes of goodwill.” Professionals’ experiences of delivering 24/7 end-of-life care to children and their families: a qualitative study**

Supplementary file 2: Theme and subthemes

| **Theme/subtheme name** | **Description** |
| --- | --- |
| Theme 1: Working within a fragmented landscape | |
| Responding to the need for 24/7 end-of-life care | Wide variation in HCP’s ability to respond to the need for 24/7 end-of-life care, with only a few teams having the infrastructure to provide seamless, trusted, and continuous support. |
| Coordination across teams without infrastructure | Coordinating care across multiple teams without shared systems or formal processes placed a heavy reliance on personal mobile numbers, timely handovers, and proactive communication. |
| HCPs building 24/7 continuity through integration | HCPs worked across organisational boundaries, through shared rotas, co-location, dual roles, and local skill-building to create more sustainable and integrated 24/7 care models. |
| Theme 2: Constraints on choice: default not preferred choice? | |
| Limits to family choice | While professionals aimed to support families' preferences for end-of-life care, genuine choice was often constrained by service availability, resource limitations, and local infrastructure. |
| Critical yet inconsistent provision of community nursing | The capacity of community children’s nursing teams to deliver end-of-life care varied widely within regions, significantly influencing where and how children could be cared for. |
| Inequality of access to specialist support | Access to timely, expert palliative care support, especially out-of-hours, was inconsistent leaving families and healthcare professionals without the guidance and reassurance they needed. |
| Theme 3: The personal cost of making it work | |
| Gaps in confidence and experience | HCPs identified gaps in confidence, training, and hands-on experience needed to deliver high-quality 24/7 care. |
| The price of goodwill | The toll on HCPs of sustaining 24/7 end-of-life care and concerns that their efforts masked systemic shortcomings. |
